# The Week's Work

**Published:** 1911-02-04

**Authors:** 


### The Week's Work.

### A RECORD OF PROGRESS AT LEEDS.

The Lord Mayor of Leeds has done a very useful public service in presiding over a meeting, the object of which is to raise £150,000 "for the maintenance and extension of the General Infirmary." Having published a Report on this institution in The Hospital of January 29, 1910, we are able to state that the Leeds General Infirmary deserves all the financial support that Leeds can give to it. Indeed, in the words of our Report, "anyone in Leeds who has money to spare may, with the fullest confidence, place it, or some of it, at the disposal of the treasurer and committee of the Leeds General Infirmary." The treasurer, Mr. Charles Lupton, is in the happy position of knowing that the Lord Mayor, Mr. W. Middlebrook, M.P., has received already some £70,000 towards the extension scheme. That this money has come in so generously shows that the city of Leeds has taken to heart The Hospital's prophecy, which was made in the Report above quoted, that "the time is approaching when it will be desirable, if it is not even necessary, to reconsider the whole plan of the existing buildings," and, probably, to decide on the removal of the infirmary "to a more airy and more suitable site." It is, again, satisfactory to note that Mr. Charles Lupton suggested at the meeting the carrying out, if the scheme proved a success, of a most important proposal—namely, the building of four new operating theatres. If anyone thinks this an extravagant suggestion he had better turn to our Report again, in which "the limited number of operation theatres and the extreme pressure which exists upon those theatres practically every day of the week" were two of the defects especially emphasised. It is hardly necessary to add that the reconstruction scheme is to form a memorial to King Edward. Bradford last week decided on a similar course, and we noted then the foundation of a County or West Riding Fund, the object of which is to provide money for the local hospitals as memorials in more than one place. With this Fund it was resolved at the meeting to co-operate, Leeds citizens being recommended to allocate their gifts to their own infirmary. We hope that every Yorkshire city will join in this movement, of which Bradford and Leeds are the pioneers, for Yorkshire men could have no finer or more useful memorial than a county in which every hospital had been brought up to date in grateful memory to the Royal pioneer of hospital progress.

### THE CRITIC'S REWARD.

The title of the preceding paragraph is one which recurs in "Week's Work," and its repetition is for the ready reference of readers anxious to follow the thread of hospital development in these columns. In October 1909 we began a series of Reports on the Hospitals of the United Kingdom. The paragraphs indicated by the same title record the effect of those Reports. It is not in a boastful spirit that we venture to draw our readers' attention to the reconstruction schemes now in progress all over the country; they become doubly valuable when read in the light of the Reports which preceded them. The comparison, for instance, of the proposals now maturing at Hartshill, at Bradford, at Chester, at Leeds, to take the four latest examples, with Sir Henry Burdett's Reports on these institutions, is extremely instructive. To prove that it is not vain glory which prompts this statement, we may be forgiven for adding that while reference to Reports has been given wherever new schemes have been undertaken, no reference has been made to the criticisms which sometimes greeted those Reports. Yet these criticisms make amusing reading to-day, having been often excited in the very institutions which now are so active in carrying our suggestions into effect. This activity shows that the Reports were written in a spirit of constructive criticism, for if criticism were excited by them, construction has followed in practically every case. The Hospital of October 2, 1909, p. 17, should be compared with that of November 19, 1910, p. 237, if anyone thinks that the critic is not rewarded in the end.

### DEATH CERTIFICATION IN HOSPITALS.

The recent scare raised by Dr. Icard, who is fairly well known to those who are interested in tests for establishing the fact that life is finally extinct in the organism, is much to be deprecated, for it not only gives the public an utterly false impression of what goes on in hospitals, but it raises doubts as to the validity of certain prior arguments advanced by this scientist in his fight against premature burial. We have already expressed our opinion on the necessity for a better system of death certification than exists at present, and there is, therefore, no necessity to repeat that nowadays, under the law as it stands, there is a possibility that serious dangers to the individual and the community may arise through oversight or by intention. But while we admit that, we are utterly unable to agree with Dr. Icard that in hospitals especially there exists a horrible laxity in regard to death certification. "No precautions," we are told, "are taken to ascertain whether patients declared dead are really dead. Bodies placed in the coffins have returned to life only to die again from fear; doctors own that in signing the death certificate they have never verified the ward sister's report of the deaths, and that they never make a personal inspection of the bodies." And, referring more particularly to Paris, we are treated to the outrageous statement: "As a consequence [of this want of care in the hospitals] bodies are dissected while still alive. . . . The number of people who have been killed by doctors and students in the dissecting-room is given as forty."

### AN ATROCIOUS CALUMNY.

We have no hesitation in characterising these statements, which have appeared in an English newspaper and which, we understand, have attracted some attention in this country, as absolutely false, and every hospital worker will agree with us in that expression of opinion. We need not point out to those who know anything at all about hospitals or dissecting-rooms that neither in London nor in Paris is it possible for a living person to be "anatomised" in the dissecting-room. A human body undergoes a lengthy and elaborate process of preparation before it is suitable for dissection purposes: it must be injected, hardened, preserved, and prepared, not only to enable delicate dissections to be undertaken, but also to prevent it from being a perpetual source of danger to everyone who handles it. This preparatory process lasts, on an average, six weeks, and not even the most credulous will believe that animation can be suspended for such a length of time under ordinary conditions. The fable with regard to the dissecting-room is thus self-evident; that with regard to the ward remains, but we need scarcely add that here again the hospital worker has a mass of objections to raise against Dr. Icard's glib assertions. In the first place, the hospital doctor knows every patient under his charge, and is fully aware of the condition of each. He is invariably either present at the moment when a patient dies or immediately before the actual stage of dissolution: that, indeed, is his business, and if it were not one of his chief duties there would be no need to have a registered medical man as a resident officer to a hospital. We know of no English hospital in which the medical officer gives a certificate of death without having seen and examined the body of the patient; the usual tests—which, although no single one can be termed absolutely reliable, are nevertheless trustworthy enough when taken in combination—are applied before the death is assumed. The corpse is then removed to the mortuary, and, in most large hospitals, an autopsy is made within thirty-six hours of death. At this autopsy every chance is given to the patient, every precaution is taken, and the first superficial incision is sufficient to prove to the skilled pathologist who conducts these examinations that life is extinct. As a matter of fact, the patient who is not in a hospital runs a greater risk (small as that risk is) of being "prematurely buried" than the patient who is ill in a general or special hospital.

### THE HARM OF HOSPITAL SCARES.

These points are clear enough to those who are acquainted with hospitals and their work, with the routine in the wards and the care taken in the selection of their *personnel*. But they are, unfortunately, not so obvious to the general public, and such statements as are made by Dr. Icard call for the most emphatic repudiation from everyone interested in our charitable institutions. It is true Dr. Icard's reflections concern more the Parisian than the London hospitals, but we have some knowledge, from personal experience, of hospital ways in France, and we have no hesitation in stating that these reflections are quite unjust. All the same they are likely to impress the public, and therefore to do harm, indirectly, to the hospitals. No one is likely to be generous towards an institution that is alleged callously to dissect its patients alive! Few, unfortunately again, are in a position to know that such an allegation is as absurd as the caricature of the physician drawn by Defoe. And there is a real danger that those who are zealous in drawing attention to the need of reform in the law anent death certification will make capital out of these statements and suppress the direct refutations that have already appeared in the Press. We are convinced that the majority of those who believe that in present circumstances there is some risk of premature burial, and who are working earnestly to minimise that risk as much as possible, will deprecate with us this wholesale accusation of culpable carelessness levelled against the hospitals.

### SURPRISE VISITS TO HOSPITALS.

Hospitals, like all other institutions, resent inspection in any form, but they naturally feel that the offence is exaggerated if inspection takes the form of a surprise visit. It has been found in the course of time that when notification of an inspection is supplied beforehand the sting of the inspection is removed. Yet the value of surprise visits once again received incontestable proof when Mr. Henry Baker, the Chairman of the Mexbro' and Doncaster Joint Hospital Board, unexpectedly arrived at the Conisboro' Hospital. He had been informed that the laundry was out of order, that the engineer had been unable to keep the engines going, with the result that the hospital had spent one night in darkness, and that the radiators were all wrong. Mr. Baker, accompanied by other members of the Committee, paid an unforeseen visit to the institution. They found the laundry "in a most deplorable condition," and what else they found may be judged briefly from the fact that the engineer resigned in forty-eight hours after the visit. This surprise visit, this inspection, evidently produced good results, and inspections that have no hint of surprise about them have generally no hint of surprise either in the results which they produce. Not only special preparations can be made, but the criticism of a man who says that he is coming well in advance of his visit is apt to be tame. It requires courage to be a useful inspector: how many such hope there may be nothing to find! The inspector's only chance of protecting himself is to warn the hospital authorities. The result is the worthless catalogue of insipid praise which an average visitor's book abounds in. Surprise visits must be supported if we are to have inspections that are of any value or inspectors with a criterion of their own. We are safe from impostors because the impostor cannot support his allegations in the face of the expert criticism by which hospital workers can put them to the test.

